# Long-term satisfaction and quality of life following risk reducing surgery in *BRCA1/2* mutation carriers

**DOI:** 10.1186/1897-4287-12-9

**Published:** 2014-04-02

**Authors:** Gillian W Hooker, Lesley King, Lauren VanHusen, Kristi Graves, Beth N Peshkin, Claudine Isaacs, Kathryn L Taylor, Elizabeth Poggi, Marc D Schwartz

**Affiliations:** 1 Department of Oncology, Jess and Mildred Fisher Center for Familial Cancer Research, Lombardi Comprehensive Cancer Center, Georgetown University, Washington, DC 20057, USA; 2 Department of Medicine and Oncology, Jess and Mildred Fisher Center for Familial Cancer Research, Lombardi Comprehensive Cancer Center, Georgetown University, Washington, DC 20057, USA; 3 Social and Behavioral Research Branch, National Human Genome Research Institute, National Institutes of Health, Bethesda, USA; 4 School of Medicine, University of Washington, Washington, DC USA; 5 Department of Oncology, Lombardi Comprehensive Cancer Center, 3300 Whitehaven Street, NW, Suite 4100, Washington, DC 20007, USA

**Keywords:** *BRCA1/2*, Decision making, Satisfaction, Quality of life

## Abstract

**Background:**

As *BRCA1/2* testing becomes more routine, questions remain about long-term satisfaction and quality of life following testing. Previously, we described long term distress and risk management outcomes among women with *BRCA1/2* mutations. This study addresses positive psychological outcomes in *BRCA1/2* carriers, describing decision satisfaction and quality of life in the years following testing.

**Methods:**

We evaluated satisfaction with testing and management decisions among 144 *BRCA1/2* carriers. Prior to genetic testing, we assessed family history, sociodemographics and distress. At a mean of 5.3 years post-testing, we assessed management decisions, satisfaction with decisions and, among women with cancer, quality of life.

**Results:**

Overall, satisfaction with decision making was high. Women who had risk reducing mastectomy or oophorectomy were more satisfied with management decisions. Participants who obtained a risk reducing oophorectomy were more satisfied with their genetic testing decision. Among affected carriers, high pretest anxiety was associated with poorer quality of life and having had risk reducing mastectomy prior to testing was associated with better quality of life. The negative impact of pre-test anxiety was diminished among women who had mastectomies before testing.

**Conclusions:**

*BRCA1/2* carriers are satisfied with their testing and risk management decisions and report good quality of life years after testing. Having risk reducing surgery predicts increased satisfaction and improved quality of life.

## Introduction

Decisions about genetic testing and medical management among women at increased risk for breast and ovarian cancer are complex and driven by a combination of medical recommendations and patients’ value-based preferences. As *BRCA1/2* testing has become routine, increasing numbers of women now face these decisions. Women who carry mutations in the *BRCA1* or *BRCA2* (*BRCA1/2*) gene have a lifetime risk of 40% to 75% of developing breast cancer and up to a 40% risk of developing ovarian cancer [1]. To manage these risks they must choose between increased surveillance and risk reducing surgery. Increased surveillance may involve annual mammography and MRI for breast cancer and ultrasonagraphy and CA-125 blood tests for ovarian cancer. Alternatively, surgical options include risk reducing mastectomy (RRM) and oophorectomy (RRO). Current guidelines recommend RRO between the ages of 35 and 40 or upon completion of childbearing and that RRM be discussed as an option on a case-by-case basis [2,3].

Several recent studies document that the majority of *BRCA1/2* mutation carriers opt for RRO, RRM or both [4-10]. Thus, it is critical to understand long-term patient satisfaction and quality of life following these surgeries. Although prior studies have indicated that patients typically report high satisfaction with these decisions [11-15], few have prospectively compared *BRCA1/2* carriers who did and did not opt for risk reducing surgery. One study, using single item assessments of satisfaction, found no difference in satisfaction associated with risk reducing mastectomy [12]. Other identified correlates of satisfaction include family history, cancer risk, cancer worry and age [12,13,16]. Of note, the populations in these studies were selected based on family history rather than test result, and thus had variable levels of cancer risks. As such, it is difficult to separate the possibly confounding effect of risk on uptake of risk reducing surgery and on satisfaction post-surgery. In terms of RRO, Madlinska and colleagues evaluated satisfaction using a series of single item questions in a large cohort of women (43% of whom were *BRCA1/2* mutation carriers) who had either undergone RRO or gynecologic screening a mean of four years following a high risk gynecology visit [17]. In this study, women who had opted for RRO were more satisfied than those undergoing regular gynecologic screening, leading the authors to suggest that oophorectomy may be not only medically advantageous, but also psychologically advantageous for many of the women in their study.

Despite the apparent high rates of satisfaction with risk reducing surgery decisions, it remains unclear if such surgery significantly impacts *BRCA1/2* carriers’ long-term quality of life. A short-term negative impact of surgery on physical functioning domains of quality of life has been noted, most likely related to recovery from surgery [18], but most other studies of high risk women suggest comparable or higher quality of life scores over time, in association with RRO and RRM [12,17,19-21]. All of these studies followed women at varying levels of genetic risk and various time points (12 months-10 years) following risk assessment and/or surgery. None of these studies were designed to control for the potential confounding effect of women making decisions about both RRM and RRO.

While some high-risk women opt for surgery very soon after learning their *BRCA1/2* mutation status, others make these decisions over time [4]. As such, long-term prospective studies are necessary to gain a more complete understanding of the impact of *BRCA1/2* testing and subsequent decision-making. Previous reports, including one from this cohort, have addressed long term distress outcomes and found that *BRCA1/2* carriers experience distress related to their genetic status that is significantly greater, though not clinically significant, compared to those who do not carry *BRCA1/2* mutations [22,23]. Questions remain regarding positive outcomes related to undergoing testing and the decisions made following testing. This study is the first to prospectively examine long-term satisfaction and quality of life outcomes in a U.S. cohort of *BRCA1/2* carriers with and without a history of cancer. In this report, we describe decisional satisfaction over time and long-term quality of life, comparing women who have made different decisions regarding the management of their breast and ovarian cancer risk.

## Methods

### Ethics statement

This study was approved by the Georgetown University Institutional Review Board (Protocol #2003-004, “Long Term Outcomes of BRCA1/BRCA2 Testing”) and the USAMRMC Office of Research Protections (Protocol #A-12074). Written informed consent was obtained from all participants on Institutional Review Board-approved consent documents.

### Study population

We recruited participants from the Lombardi Comprehensive Cancer Center (LCCC) familial cancer registry (FCR). All participants had received clinical genetic counseling and testing at LCCC, were at least 3 years post-disclosure of *BRCA1/2* test results and had participated in one of four previous studies: two observational studies of the short-term outcomes of genetic counseling and testing [5,24,25] and two post-test intervention trials in *BRCA1/2* carriers, one of a psychosocial telephone counseling intervention [26] and one of a computer-based decision aid designed to assist *BRCA1/2* carriers in making decisions about managing their breast cancer risk [27]. All participants completed a baseline interview prior to pre-test genetic counseling, from which data are included in the present study. The individuals randomized to receive interventions did not differ significantly on any of the outcomes addressed in the analyses in this study.

The present report is limited to *BRCA1/2* mutation carriers. Of 206 potentially eligible *BRCA1/2* carriers who were active in the FCR, we had incorrect contact information for 9. Of the remaining 197 eligible women, 38 (19.2%) declined participation and 15 (7.6%) could not be reached after repeated attempts. Our final sample (N = 144) represents 73.1% of those who were eligible and for whom we had correct contact information. Participants differed from eligible non-participants on three sociodemographic variables. Participants were more likely to be White (p = .03), Jewish (p < .001) and college graduates (p = .003). Participants and decliners did not differ on any psychosocial or personal/family history variables.

### Procedures

We mailed eligible participants study packets containing a study invitation, Institutional Review Board approved consent documents, a printed version of the survey, a stamped return-addressed card to decline participation, and a telephone number to call should they wish to provide their answers by phone, or decline by phone.

## Measures

### Control and predictor variables

#### Sociodemographics

We measured age, race/ethnicity, education, marital status, employment status and religion.

#### Time since receipt of genetic test result

We calculated the time elapsed between the date that participants received their *BRCA1/2* result and the date they completed the current survey.

#### Personal and family cancer history

We assessed participants’ personal history of breast and gynecologic cancer and time since diagnosis. For logistic regressions, we divided the time since diagnosis variable into 3 groups: unaffected women, those diagnosed less than 10 years ago and those diagnosed more than 10 years ago. We also assessed the number of first- and second-degree relatives with breast and/or ovarian cancer.

#### Pre-counseling anxiety

Our measure of baseline anxiety is a composite of two anxiety measures we used in previous studies. Twenty-three participants completed the 20-item State Anxiety subscale of the State Trait Anxiety Inventory (STAI) [28] and 121 participants completed the anxiety subscale of the Brief Symptom Inventory (BSI) [29]. As in previous reports [4,22], we used each participant’s z-score on the measure they completed as their measure of pre-counseling anxiety. Both measures are reliable (Cronbach’s alpha = 0.85 (STAI) and 0.91 (BSI)).

#### Risk-reducing surgery

Using face-valid interview questions previously described [4], we assessed receipt of risk-reducing mastectomy (RRM ever), oophorectomy (RRO ever), time elapsed since each surgery, and the timing of surgery relative to genetic testing (pre-testing, post-testing). Women who had mastectomy or oophorectomy for reasons other than prevention (e.g. bilateral breast cancer or ovarian cysts) were excluded from analyses applying these variables.

### Outcome variables

#### Satisfaction with management and genetic testing decisions

All participants completed modified versions of the reliable and valid Satisfaction with Decision Making (SWD) scale [30]. We modified the SWD for the present study by eliminating a single item, which was phrased “I expect to successfully carry out (or continue to carry out) the decision I made”. We deleted this item because genetic testing, RRO and RRM are one-time decisions. Thus, this item was not applicable to those who had already obtained either surgery. Previous research has successfully employed modified versions of this scale [31]. Participants completed the 5-item modified SWD scale for satisfaction with genetic testing (alpha = 0.92) and for satisfaction with management decisions (alpha = 0.94). However, since scores on the SWD were highly skewed for both outcomes, we dichotomized the scale dividing those who were “very satisfied” across all items from those who were less than “very satisfied” on one or more items.

#### Quality of life

We assessed quality of life among the affected women in our sample (N = 97) using the 36-item FACT-B (Functional Assessment of Cancer Therapy- Breast) designed for use in breast cancer patients (alpha 0.89) [32].

### Statistical analysis

We characterized the sample in terms of sociodemographics, personal history of cancer, cancer treatment and prevention decisions, anxiety prior to genetic testing and time since genetic testing. We used chi-square and *t*-tests (with Satterthwaite’s approximation for unequal variances when appropriate) to identify bivariate predictors of satisfaction with management decisions, satisfaction with genetic testing and, among women with a history of cancer, quality of life. Next, we used logistic (for satisfaction with management decisions and satisfaction with the genetic testing decision) and linear regression (for quality of life) with hierarchical variable entry in which we included all main effect variables with p < 0.10 bivariate associations with the outcome of interest on the first step. Because quality of life had multiple independent predictors, we conducted follow-up testing for interactions between the main effects by entering the interaction term on the second step of the linear regression model. For models of satisfaction with management decisions, we excluded women who had bilateral mastectomies or oophorectomies for reasons other than prevention. For all models, when testing variables with significant colinearity (e.g. RRM ever, RRM before genetic testing and time since RRM; RRM before genetic counseling and time since RRM) we included only the variable with the stronger bivariate association.

## Results

### Sample characteristics

As displayed in Table 1, 90 of the 144 (62.5%) *BRCA1/2* mutation carriers in this sample had a prior diagnosis of breast cancer, five had been diagnosed with ovarian cancer, one had both breast and ovarian cancer and one had breast and uterine cancer. On average it had been a mean of 5.2 (SD = 1.2) years since genetic testing. Among affected women, it had been a mean of 10.9 (SD = 7.9) years since their diagnosis. The majority of participants were white (94%), college educated (96%), and married (77%). The mean age of participants at long-term follow up was 49.6 (SD 10.0), and they had a mean of 2.8 first or second-degree relatives with breast or ovarian cancer.

**Table 1:** Characteristics of the study sample

**Cancer history**	
Breast cancer^*a*^, N (%)	90 (62.5)
Ovarian cancer, N (%)	5 (3.5)
Both breast and ovarian, N (%)	1 (0.7)
Both breast and endometrial, N (%)	1 (0.7)
Unaffected, N (%)	47 (32.6)
Time since cancer diagnosis, mean (SD)	10.9 (7.9)
Years since testing, mean (SD)	5.2 (1.2)
Age at F/U, mean (SD)	49.6 y (10.0)
Range	32y-76y
College degree or higher, N (%)	139 (96.5)
Jewish, N (%)	48 (33.3)
White, N (%)	135 (93.8)
Married, N (%)	111 (77.1)
Relatives with cancer, mean (SD)	
Breast cancer	2.3 (1.2)
Ovarian cancer	0.5 (0.7)
Breast or ovarian cancer	2.8 (1.5)
Oophorectomy	
Risk reducing oophorectomy^*b*^ N (%)	94 (65.2)
*Prior to genetic testing,* N (%)	*29 (20.1)*
*After genetic testing*, N (%)	*65 (45.1)*
For treatment of ovarian cancer, N (%)	6 (4.2)
Other^*c*^, N (%)	10 (6.9)
Ovaries intact, N (%)	34 (23.6)
Years since risk reducing oophorectomy, mean (SD)	5.49 (4.18)
Mastectomy	
Risk reducing mastectomy^*d,e*^, N (%)	64 (44.4)
*Prior to genetic testing*, N (%)	*24 (16.6)*
*After genetic testing*, N (%)	*40 (27.8)*
Bilateral for treatment, N (%)	13 (9.0)
Unilateral for treatment, N (%)	12 (8.3)
Both breasts intact, N (%)	55 (38.2)
Years since risk reducing mastectomy, mean (SD)	6.35 (6.3)
Satisfaction with management decisions, mean (SD)	23.4 (2.87)
Very satisfied with management decisions, % (number)	65.3% (94/144)
Satisfaction with genetic testing decision, mean (SD)	23.6 (2.56)
Very satisfied with genetic testing decisions, % (number)	66.0% (93/141)
Quality of life, FACT-B for affected women, mean (SD)	117.0 (19.0)
Quality of life, SF-12 for unaffected women, mean (SD)	100.8 (14.0)

^*a*^4 women with breast cancer were still undergoing chemotherapy ^*b*^27/47(57%) unaffected women, 67/90 (74%) women with breast cancer ^*c*^(ovarian cysts-7, endometrial ca-2, menstrual discomfort-1) ^*d*^Includes both women who had bilateral prophylactic mastectomy and women who had contralateral prophylactic mastectomy ^*e*^17/47 (36%) unaffected women, 47/97 (48%) women with any cancer history.

As described in our previous report [4,22], the majority of participants had undergone RRO and almost half had obtained a RRM at the long term follow up.

### Satisfaction and quality of life

Participants reported extremely high satisfaction with their decision to undergo *BRCA1/2* testing and with their management decisions following receipt of a positive test result. Over 90% of participants scored a 20 or higher (out of 25) on both satisfaction scales and over 60% of participants scored a perfect 25 on both scales.

In bivariate analyses of satisfaction with the genetic testing decision (Table 2), high satisfaction, or being “very satisfied” across all items on the SWD scale, was associated with: ever having RRM (***Χ***^**2**^ (1, N = 126) = 12.5, p < 0.001), having had RRM before testing (***Χ***^**2**^ (1, N = 126) 16.04, p < 0.001), and a shorter time since cancer diagnosis (*r*(94) = −0.26, p < 0.05).

**Table 2:** Bivariate predictors of satisfaction and quality of life

**Variable**	**High satisfaction with management decisions**	**High satisfaction with genetic testing**	**Quality of life (FACT-B)**
	***Percent (N)***	***Percent (N) SD***	***Mean (N) SD***
Affected status			
*Unaffected*	62% (29)	57% (27)	N/A
*Prev. cancer diagnosis*	67% (65)	70% (66)	116.98 (96) 18.99
Oophorectomy			
*Ovaries intact*	53% (18)^*^	58% (19)	123.64 (17) 2.83
*Risk Reducing Oophorectomy (RRO)*	74% (91)	72% (64)	117.67 (64) 2.28
Mastectomy			
*≥1 at risk breast*	55% (36)^*^	55% (35)^**^	119.46 (35) 15.11
*Risk Reducing Mastectomy (RRM)*	77% (49)	84% (52)	118.99 (48) 17.22
Timing of RRM			
*Pre-testing*	88% (21) 1.50^*^	88% (21)^**^	124.28 (22) 11.04^*a^
*Post-testing*	70% (28) 2.77	82% (31)	114.52 (26) 20.24
*Never*	55% (36) 2.89	55% (35)	114.98 (48) 20.57
Timing of RRO			
*Pre*	85% (21) 0.88^*^	69% (18)	116.37 (17) 22.11
*Post*	69% (45) 2.51	73% (46)	118.15 (47) 16.81
*Never*	53%(18) 3.47	58% (19)	123.64 (17) 11.68
Continuous variables	*r*	*r*	*r*
Age	0.03	−0.06	−0.01
Time since testing	−0.08	−0.05	0.01
Time since diagnosis	−0.16	−0.260^*^	<0.01
Time since mastectomy	0.24^	0.11	0.322^*^
1st degree relatives w/ breast or ovarian cancer	−0.04	−0.05	0.01
Pretest anxiety	−0.03	0.08	−0.313^**^

^p < 0.10 ^*^p < 0.05 ^**^p ≤ 0.001, ^*a*^compared to women without this prophylactic surgery.

High satisfaction with management decisions, also assessed as scoring a “very satisfied” across all items on the SWD scale, was associated with: having ever received an RRM (***Χ***^**2**^ (1, N = 129) = 6.44, p = 0.01) and having ever received an RRO (***Χ***^**2**^(1, N = 125) = 4.87, p = 0.03). Timing of RRM and RRO was also associated with satisfaction. Women who underwent RRM prior to testing were more likely to report high satisfaction with their management decisions (***Χ***^**2**^(1, N = 129) = 8.50, p = 0.01) and women who had RRO prior to testing were also more likely to report high satisfaction (***Χ***^**2**^(1, N = 125) = 6.89, p = 0.03). Notably, having had a diagnosis of cancer was not significantly associated with satisfaction with management decisions or with genetic testing satisfaction.

In terms of quality of life among women with a history of cancer, greater time since RRM (among affected women who had RRM) was associated with higher quality of life (*r*(48) = 0.32, p = 0.03) and high pretest anxiety was associated with lower quality of life (r(96) = −0.31, p = .002). Although ever having received RRM or RRO was not associated with quality of life, timing of RRM was significantly associated. Women who underwent RRM prior to testing reported significantly higher quality of life at follow-up compared to those who had never undergone RRM (t(68) = −1.98, p = .05) and those who underwent RRM after testing (Satterthwaite t(39.8) = 2.11, p = 0.04).

### Multivariate models of satisfaction and quality of life

#### Satisfaction with genetic testing decision

To identify independent predictors of high satisfaction with the genetic testing decision, we conducted a multiple logistic regression analysis including all variables with a bivariate association with genetic testing satisfaction at a significance level of p < 0.10 (RRM Ever, RRM before testing and time since diagnosis). Because RRM ever and RRM before testing were highly correlated, we included only RRM ever in the multivariate model due to its stronger bivariate association. In order to include unaffected women in the model, we created a dummy coded variable for time since diagnosis in which unaffected women were the referent generating comparisons with women diagnosed less than 10 years ago and with women diagnosed more than 10 years ago. Neither the omnibus time since diagnosis variable (p = 0.31) nor the constituent comparisons (Table 3) were significant in the multivariate model. Ever having received RRM was the only independent predictor of high satisfaction with genetic testing (OR = 4.3, 95% CI: 1.9-9.9).

**Table 3:** Multivariate predictors of satisfaction and quality of life

**Variable**	**OR**	**95% CI**	***p***		
**Satisfaction with genetic testing decision**					
Step 1	4.3	1.9-9.9	0.001		
RRM					
Step 2					
Time since diagnosis			0.31		
Unaffected (Referent)	1.0	--	--		
Less than 10 years since diagnosis	2.0	0.8-5.2	0.16		
More than 10 years since diagnosis	1.1	0.4-2.8	0.92		
**Satisfaction with management decisions**					
RRO	2.4	1.0-5.5	0.04		
RRM	2.4	1.0-5.8	0.05		
**Quality of life (FACT-B)**					
Variable	Step 1 β	Total *R*^*2*^	Δ*R*^*2*^	df	Step 2 β
Step 1		0.16***			
Baseline anxiety	−0.34***				−0.49***
RRM before testing	0.24*				0.21*
Step 2		0.20***	0.04*	3,92	
Anxiety X RRM before testing					0.26*

*p < 0.05 , **p < 0.01, ***p < 0.001.

#### Satisfaction with management decision

We used the same approach to identify independent predictors of high satisfaction with management decision. As in the previous model, candidate variables were overlapping (e.g., RRO ever and RRO before genetic testing; RRM ever and RRM before genetic testing and Time Since Mastectomy). In each case we chose the variable with the stronger bivariate association with satisfaction. Given that our questions were anchored on management of ongoing cancer risk vs. decisions about cancer treatment, and the lack of significant association between cancer history and satisfaction, we included both unaffected women and women with a history of cancer in our model. Thus, our multivariate model included RRO ever and RRM ever. As displayed in Table 3, both receipt of RRO (OR = 2.4, 95% CI: 1.0-5.8) and RRM (OR = 2.4, 95% CI: 1.0-5.5) independently predicted high satisfaction with management decisions.

#### Quality of life

We used linear regression to identify independent predictors of long-term quality of life among women with a personal history of cancer (n = 97). As above, we included variables with bivariate association of p < 0.1 with quality of life: baseline anxiety and RRM prior to testing, excluding time since mastectomy because of its overlap with RRM prior to testing. Higher baseline anxiety (β = −0.34, p < 0.001) independently predicted poorer long-term quality of life and RRM prior to genetic testing (β = −0.24, p = 0.01) independently predicted better long-term quality of life (Table 3). We also identified a significant interaction between RRM prior to testing and anxiety (β = 0.26, p = 0.03). Stratified analyses indicated that, among women who had RRM prior to testing, baseline anxiety did not predict quality of life (r = −0.14, p = 0.55) but among women who had not had an RRM prior to testing, baseline anxiety predicted poorer quality of life (r = −0.42, p <0.001).

## Discussion

This prospective study provides new insights into long-term satisfaction and quality of life in a U.S. cohort of *BRCA1/2* mutation carriers. Consistent with previous studies [11-13,17,21,33-35], satisfaction with both the decision to have genetic testing and with specific management decisions was extremely high. Overall, more than 60% of the sample reported perfect satisfaction with both the decision to undergo genetic testing and their management decisions following the receipt of genetic testing results. Given the increasing use of genetic testing, these data should reassure clinicians and patients regarding the long-term impact of genetic testing and subsequent medical decision-making.

Although overall satisfaction was extremely high, we did find that women who opted for RRO or RRM were significantly more satisfied with their management decisions than women who had not had these surgeries. This is among the first studies to examine RRO and RRM decisions concurrently within the same sample. These analyses add to the limited evidence suggesting that long-term satisfaction is higher among women who opt for these risk reduction surgeries compared to those who do not [17].

Longitudinal studies such as this one provide important insight into the potential impact of cancer risk assessment and risk reduction over time. Our finding that, among affected women, time since surgery and having had RRM before testing are both associated with higher quality of life supports the idea of a long-term benefit that may increase over time. Though we were not powered to detect an effect of time since surgery in our multivariate model of quality of life, the significant effect of RRM prior to testing may be due to the fact that these women also had a greater time since surgery. Women who had an RRM more recently may still be burdened by problems with reconstruction and recovery. Alternatively, it may be that the decision to receive risk reducing surgery prior to testing reflects an especially strong preference for risk reducing surgery; a preference subsequently validated by the receipt of a positive test result. In contrast, women who did not obtain risk reducing surgery or opted for such surgery after receiving a positive test result may have had more ambivalence about the decision, leading to poorer long-term quality of life.

Interestingly, having had an RRM before testing modified the relationship between baseline anxiety and subsequent quality of life. Baseline anxiety did not predict quality of life among women who had an RRM prior to testing but predicted poorer quality of life for those who had not had an RRM prior to genetic testing. This finding could reflect a response shift among women who had a RRM prior to testing. Response shift refers to a recalibration, reprioritization or reconceptualization of quality of life over time following an impactful event [36]. Perhaps women who had an RRM prior to testing have had more time living with the impact of their surgery and as a result are more likely to exhibit response shift. Thus, their anxiety at the start of the study may be less relevant to their current recalibrated quality of life. Women who had not obtained an RRM or who obtained one more recently would be less likely to have exhibited a response shift. Another plausible explanation is that among women who had an RRM at the time of the initial assessment, self-reported anxiety may not have been strongly influenced by concerns about their risk (which had already been greatly reduced due to their RRM). Thus, the more general anxiety they reported at that time would not be expected to prospectively predict their quality of life. In contrast, the anxiety among women who had not had RRM may have more strongly reflected their concerns about risk. Their current quality of life may reflect the same anxiety associated with their ongoing burden of living at risk.

The findings reported in this study should be interpreted in the context of its limitations. First, the study sample is comprised exclusively of clinical research participants seen at a single institution. Consistent with other studies conducted at large cancer genetics referral centers, study participants were in general, highly educated with limited racial diversity. Second, we drew from data collected from participants prior to genetic testing (baseline) and at long-term follow up and propose that difference in time between reference points (testing, surgery, diagnosis) reflect progress of our outcomes over time. It is also possible that changes not measured or controlled for in the intervening time periods (e.g. in the clinical care and follow up of study participants) could confound the interaction effect between time since testing and satisfaction with management decisions. We modified the Satisfaction with Decision scale for this study, to make it more appropriate for the risk management decisions addressed in this study. Additionally, there may be other differences in variables not assessed in this study between the group of women who underwent RRM and those who did not that might contribute to the differences in outcomes between these groups. Though our outcomes did not differ with regard to women’s cancer history, and the focus of this paper is on risk management, rather than disease management, we were underpowered to do subgroup analyses of affected and unaffected women, and cannot rule out cancer treatment variables as potential confounders. Finally, as testing has become more routine, there are likely to be fewer women who opt for risk reducing surgery prior to genetic testing – making our results regarding timing of surgery less relevant to the current clinical setting.

## Conclusions

This study is the largest prospective report of long-term outcomes related to surgical decision making in affected and unaffected *BRCA1/2* carriers. We provide evidence to support long-term impacts of the decision to undergo mastectomy and oophorectomy, and the lasting effects these decisions have as time increases. It is clear, from this study and others, that receiving a positive test result is not an isolated stressor, and that there are many ongoing stressors and decisions to be faced over time. As such, long-term outcomes reflect the combined impact of these multifaceted experiences. Indeed, in our previous study from this population, RRM and RRO were associated with decreased perceived risk for breast and ovarian cancer [4]. Furthermore, both were associated decreased distress related to the test result [22]. Though these results are consistent with our findings, this study expands insight into the range of outcomes, positive and negative, experienced by women following testing. These data can inform decision support provided to women as they continue to make their decisions over time, and also the development of targeted interventions to help women reach decisions that are most satisfying to them. It is critical that we recognize that living at increased risk for cancer is not a single event or stressor, and that there may be utility in continued support over time for individuals at significantly increased risk. Additionally, these data can provide some reassurance for women undergoing surgery that most women are satisfied with their decision and that their satisfaction and possibly their overall quality of life seem to increase over time.

## Competing interests

The authors declare that they have no competing interests.

## Authors’ contributions

GWH performed statistical analysis and oversaw drafting of the manuscript. LK and LV performed literature reviews and assisted in the drafting of the manuscript. KH, BNP and EP provide project management and oversight in participant recruitment and data management. EP conducted interviews and coordinated survey collection. KG, BNP, CI, KT participated in the design of the study and clinical recruitment of the original cohorts of participants. MDS conceived of the study, participated in design and coordination, oversaw the statistical analysis and helped to draft the manuscript. All authors read and approved the final manuscript.
